# Peptides in headlock – a novel high-affinity and versatile peptide-binding nanobody for proteomics and microscopy

**DOI:** 10.1038/srep19211

**Published:** 2016-01-21

**Authors:** Michael B. Braun, Bjoern Traenkle, Philipp A. Koch, Felix Emele, Frederik Weiss, Oliver Poetz, Thilo Stehle, Ulrich Rothbauer

**Affiliations:** 1 Interfaculty Institute of Biochemistry, Eberhard-Karls University Tuebingen, Germany; 2 Pharmaceutical Biotechnology, Eberhard-Karls University Tuebingen, Germany; 3 Natural and Medical Sciences Institute at the University of Tuebingen, Reutlingen, Germany; 4 Vanderbilt University School of Medicine, Nashville, TN, USA

## Abstract

Nanobodies are highly valuable tools for numerous bioanalytical and biotechnical applications. Here, we report the characterization of a nanobody that binds a short peptide epitope with extraordinary affinity. Structural analysis reveals an unusual binding mode where the extended peptide becomes part of a β-sheet structure in the nanobody. This interaction relies on sequence-independent backbone interactions augmented by a small number of specificity-determining side chain contacts. Once bound, the peptide is fastened by two nanobody side chains that clamp it in a headlock fashion. Exploiting this unusual binding mode, we generated a novel nanobody-derived capture and detection system. Matrix-coupled nanobody enables the fast and efficient isolation of epitope-tagged proteins from prokaryotic and eukaryotic expression systems. Additionally, the fluorescently labeled nanobody visualizes subcellular structures in different cellular compartments. The high-affinity-binding and modifiable peptide tag of this system renders it a versatile and robust tool to combine biochemical analysis with microscopic studies.

In the post-genomic era the field of proteomics has grown dramatically. For a multitude of applications, ranging from mass spectrometry analysis to high-content imaging, affinity-based assays are indispensable. Antibodies evolved by the vertebrate immune system remain key reagents for most of those applications since they can be generated to recognize essentially any antigen with high affinity and specificity. Analysis of antibody sequences in combination with structural studies of antibody/antigen complexes have been essential in revealing how antibodies function^1^,^2^,^3^,^4^.

An attractive alternative to conventional multidomain antibodies are smaller, single domain polypeptides (referred to as nanobodies, Nbs) derived from heavy-chain-only antibodies of camelids^5^,^6^. Nbs selected from recombinant libraries can be efficiently produced in heterologous expression systems and their affinities are in the range of conventional IgGs^7^,^8^. Nbs can be easily functionalized and applied in immunoassays, enzyme modulation, tracing of antigens in living cells or as capture molecules towards precipitation of protein complexes *in vivo* and *in vitro*^9^,^10^,^11^,^12^,^13^. Due to the absence of the variable light chain Nbs only possess three hypervariable loops (complementarity determining regions, CDRs) compared to six CDRs present in conventional antibodies. To compensate for the loss of CDRs, Nbs exhibit distinctive features regarding their CDR structure and antigen binding mode. In order to provide a sufficiently large antigen-interacting surface^14^ of 600–800 Å^2^, most nanobodies exhibit substantially elongated CDR3 loops^15^. In some cases, the increased flexibility of such long loops is counteracted by fastening them to the Nb core with an additional disulfide bond^16^. Moreover, an enlarged binding surface is often achieved by the formation of a convex paratope that can access cavities or clefts in the antigen^14^. Consequently, Nbs have mainly been reported to bind conformational epitopes^6^,^14^,^17^,^18^. We have recently generated Nbs against β-catenin^19^. One of these, referred to as BC2-Nb, recognizes a short linear epitope corresponding to residues 16–27 of β-catenin. In this study, we provide a detailed structural and biochemical analysis of the BC2-Nb and its interactions with the peptide epitope, hereafter referred to as BC2 tag (BC2T). Our data reveal an unusual binding mechanism mediated by the extended CDR3 and the framework of BC2-Nb, which we term “headlock binding”. Based on the high-affinity binding properties of the BC2-Nb we developed and characterized a novel BC2T purification and detection system. We show that the immobilized BC2-Nb efficiently captures and purifies BC2-tagged proteins in their natively folded state from bacterial and mammalian cell extracts. By co-localization analysis, we show for the first time the detection of peptide-tagged cellular proteins using a fluorescently labeled nanobody. This versatile capture and detection system now enables a unique combination of biochemical and microscopic analyses of a large variety of proteins comprising an inert peptide tag.

## Results

### Structural basis of epitope binding

Since most of the described Nbs have conformational epitopes, we investigated the molecular mechanism underlying the observed high-affinity binding by solving high-resolution crystal structures of BC2-Nb alone (2.0 Å) and in complex with BC2T (1.0 Å). From here on, we will refer to BC2T amino acids in one-letter code and to BC2-Nb amino acids in three-letter code in order to facilitate the presentation and discussion of our results.

**Figure 1:**
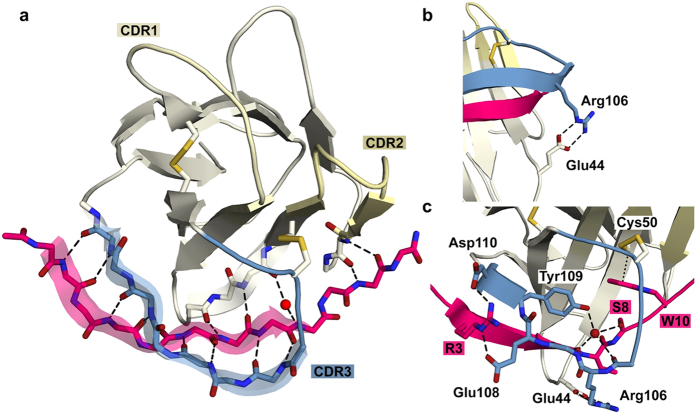
Structural analysis of the BC2-Nb/BC2T complex. (**a**) BC2-Nb/BC2T backbone interactions. The BC2-peptide (BC2T) (pink) folds into a β-strand that is part of a β-sheet structure formed by the complementarity determining region 3 (CDR3, blue) and framework regions 2 and 3 (FR2 and FR3, grey). Shown are 13 backbone-backbone hydrogen bonds (black dashed lines) of which one is mediated by water (red sphere). The CDR3 contributes eight and the framework regions five of these interactions. The CDR1 (light yellow) and CDR2 (yellow) are not participating in binding. (**b**) “Headlock” interaction. A charge-mediated interaction between Arg106 of the CDR3 (blue) and Glu44 in FR2 is stabilizing the BC2-Nb/BC2T complex. These amino acid side chains are reaching over the peptide (pink), forming a salt bridge that locks the peptide into its binding site. (**c**) Specific BC2-Nb/BC2T interactions. In addition to the backbone interactions, a small number of interactions mediated by side chains generate specificity for the BC2T peptide sequence. BC2T residue W10 is involved in a CH-π interaction with Cys50 (dotted line). A water (red sphere) is bound by peptide S8 (pink), Tyr109, two carbonyl groups and one amine group. The charge-mediated interaction between Arg106 (CDR3) and the side chain of Glu44 (FR2), reaching over the peptide, is also shown.

The BC2-Nb adopts the typical immunoglobulin fold, which consists of nine β-strands forming two β-sheets connected by loops and by a conserved disulfide bond between Cys22 and Cys96 (Supplementary Fig. 1a). Similar to other nanobody structures, BC2-Nb features an especially long CDR3, which contains 14 amino acids and is stabilized by an additional disulfide bond formed between Cys102 (located in the CDR3) and Cys50 (located in the framework region 2 (FR2)) (Supplementary Fig. 1a). The BC2T binds to BC2-Nb in an elongated conformation, inserting into a groove between the CDR3 and the FR2 and FR3 of BC2-Nb. The peptide is integrated into the BC2-Nb structure, forming a strand in a β-sheet (Fig. 1a). This results in a large number of backbone hydrogen bonds that anchor the peptide to neighboring secondary structure elements. Notably, neither CDR1 nor CDR2 are involved in the interaction with BC2T (Fig. 1a, Supplementary Table 1).

The unbound and bound BC2-Nb structures are similar and can be superimposed with a small overall RMSD of 0.4 Å (Phenix structure_comparison). The CDR3 loops in particular have similar orientations, with the exception of two amino acids (Arg106 and Tyr107) that are flipped almost 180 degrees (Supplementary Fig. 1b). In the unliganded BC2-Nb, the β-carbon of Arg106 is oriented towards the core structure of the Nb, while the Arg106 side chain interacts with the backbone carbonyl group of Glu108 and with the π-electron system of Tyr109. The β-carbon of Tyr107 is pointing away from the Nb and the aromatic ring is involved in a cation-π interaction with Arg45. In the complex, residues Arg106 and Tyr107 are flipped, as their β-carbons and their side chains face into opposite directions. Arg106 is now involved in a charge-mediated interaction with the side chain of Glu44 located in FR2 (Fig. 1b). This unusual interaction, which we term “headlock”, reaches over the bound peptide and locks it firmly in place. Several direct and water-mediated interactions fasten the peptide in its binding site (Fig. 1c). However, only a small subset of BC2T residues are involved in these contacts: R3 forms a salt bridge with Asp110, S8 is engaged in a hydrogen bond with the carbonyl group of Lys103 and also complexes a water molecule together with Tyr109, and W10 is buried in a hydrophobic pocket where it forms both a CH-π interaction with Cys50 and a hydrogen bond with the carbonyl group of Cys102 (a complete overview of all interactions is shown Supplementary Fig. 2).

Using the crystal structures as a guide, we investigated the impact of point mutations on the binding properties of the BC2-Nb. We first removed the additional disulfide bond that connects CDR3 and FR2 by replacing Cys50 with an alanine and Cys102 with a serine (BC2-Nb_C50A___C102S_). Binding studies using modified GFP that contains BC2T as a C-terminal peptide tag (GFP_BC2T_) revealed that this mutation leads to complete loss of binding (data not shown). The most likely explanation for this result is that the disulfide bridge is required to maintain the structure of the CDR3, which contributes most of the contacts with BC2T. Next, we analyzed the contribution of the “headlock” motif towards binding. We replaced Arg106 with either a serine (BC2-Nb_R106S_) or a glutamate (BC2-Nb_R106E_) and performed surface plasmon resonance (SPR) measurements using the GFP_BC2T_ construct. Both mutant proteins yield K_D_ values of ~11 nM which are about 10-fold lower compared to BC2-Nb (K_D_: 1.4 nM) (Table 1, Supplementary Fig. 3). Interestingly, the wt and mutant proteins showed similar on-rates, whereas we observed significantly higher off-rates in both mutants (Table 1). In summary, the modest changes in the affinities are consistent with the structural analysis. The thirteen backbone interactions of the extended peptide clearly contribute to the high affinity of the interaction. The “headlock” most likely helps to secure the bound peptide, as indicated by the lower dissociation rate of the wt protein.

### Detailed epitope analysis using synthetic positional scanning peptide libraries

**Figure 2:**
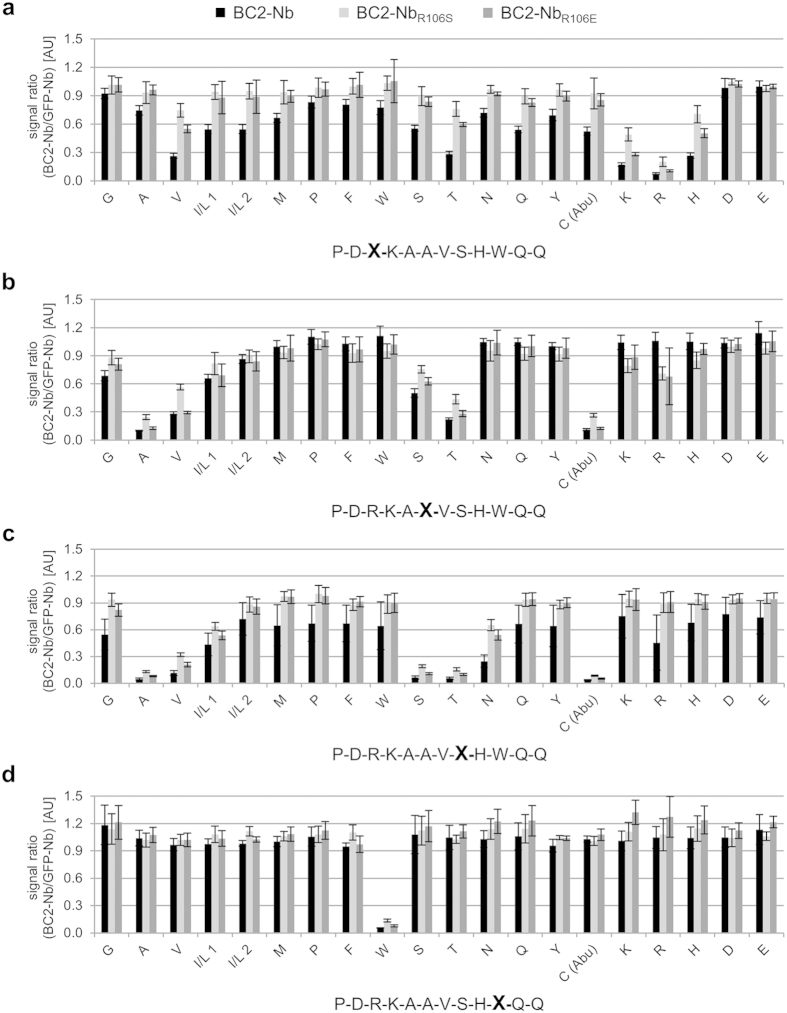
Identification of residues that mediate BC2T binding specificity. BC2T positional sequence variant libraries were incubated with BC2-Nb (black bars), BC2-Nb_R106S_ (light grey), and BC2-Nb_R106E_ (dark grey) immobilized on sepharose (n = 3). After precipitation the supernatants were subjected to liquid chromatography followed by mass spectrometry analysis. Specifically precipitated sequence variants were identified by forming a signal ratio raised by the peptides in the supernatant of BC2-Nb or indicated mutants and the supernatant of a non-BC2T-related control Nb (GFP specific Nb). Shown are results for the sequence variant libraries of (**a**) BC2T_R3X_, (**b**) BC2T_A6X_, (**c**) BC2T_S8X_, and (**d**) BC2T_W10X_. Varied positions are indicated as an X in the BC2T sequence. Amino acids are grouped according to the physicochemical property of their side chain: non-polar, polar, and charged. Gamma-amino butyric acid was used instead of cysteine in the library synthesis. Means and s.d. (error bars) of three independent experiments are shown.

Although the high-affinity binding and kinetics can be explained by the observed structural features, the specificity of complex formation must lie elsewhere. As only a subset of BC2T residues are involved in specific, side-chain-mediated contacts with the Nb, we probed the contributions of these individual amino acids for binding using positional scanning peptide libraries. In total, we used 12 different BC2T libraries displaying all 20 proteinogenic amino acids in one single position of the peptide in immunoprecipitation experiments. The BC2T libraries were subjected to liquid chromatography followed by mass spectrometry analysis before and after immunoprecipitation with immobilized BC2-Nb, BC2-Nb_R106S_ or BC2-Nb_R106E_. By determining the peptides remaining in the non-bound fraction after pulldown, we were able to map the composition of non-bound peptides and correspondingly, the invariable amino acid positions (Supplementary Table 2). Specifically precipitated peptides were identified by a comparative analysis of the peptides in the supernatant of BC2-Nb or mutants thereof and the supernatant of a non-BC2T-related control Nb (GFP-specific Nb). The direct comparison with the GFP-Nb allowed us to determine the quantitative degree of peptide capture without the need of labeled standards. Thus, if no peptide was captured by the BC2-Nb, the ratio given in Fig. 2 would be one or close to one. The more peptide was captured the more the value approximates 0. It was not possible to discriminate between isoleucine (I) and leucine (L) peptide versions since these peptides are isobaric. However, we could resolve two peptide versions containing I/L and thus two values are given as result.

Our analysis shows that BC2T residues at positions 1, 2, 4, 5, 7, 9, 11, and 12 can be replaced without affecting the epitope binding properties (Supplementary Table 2). These results are in agreement with the structure, which shows that the side chains of all eight residues face away from the Nb. However, the analysis of the supernatants of the R3, A6, S8 and W10 BC2T libraries revealed little cross-reactivities of the nanobodies to other amino acids at these positions (Fig. 2a–d). Notably, bound peptides from the R3 BC2T library (BC2T_R3X_) have either residues with basic properties, i.e. K3 or H3 or residues comprising smaller side chains, i.e. T3 or V3 (Fig. 2a). This observation is in agreement with the crystal structure as the salt bridge between R3 and Asp110 could possibly also be formed by H3 or K3. The analysis of the BC2T_A6X_ library revealed preferences of the BC2-Nb to small amino acids at position 6, as cysteine, valine, threonine and serine permutants were efficiently pulled down from the library. Peptides containing glycine, leucine or isoleucine at this position were pulled-down in smaller amounts (Fig. 2b). These results are consistent with the crystal structure, where the A6 side chain is pointing towards a small hydrophobic pocket of the BC2-Nb. Binding of larger amino acids (i.e. leucine, isoleucine) would require conformational rearrangements, and a glycine would introduce additional flexibility. A similar effect was observed for the BC2T_S8X_-library. Alanine, cysteine, threonine and valine can replace S8, while other residues were captured to a minor degree (Fig. 2c). The hydroxyl group of S8 forms a hydrogen bond with the carbonyl group of Lys103 of the nanobody. Cysteine and threonine could engage in somewhat similar, productive interactions, while the valine could at least be accommodated. The tryptophan at position 10 appears to be an invariable specificity-determining amino acid in our peptide scanning study, as none of the W10 permutants were captured by BC2-Nb (Fig. 2d). This is in excellent agreement with the structural analysis, which shows that the W10 side chain inserts into a deep, hydrophobic pocket on the nanobody.

The results of the epitope scanning for the mutants BC2-Nb_R106S_ and BC2-Nb_R106E_ are almost identical with the BC2-Nb, with one notable exception. The analysis of BC2T_A6X_ library revealed that in contrast to BC2-Nb the mutated Nbs precipitated to a small extent also BC2T variants that carried a lysine or arginine at position 6 (Fig. 2b). This observation suggests that mutation of the headlock-forming Arg45 by Ser or Glu results in somewhat lower peptide specificity. In summary, we identified four positions in BC2T with limited or no amino acid variability. The results are in accordance with the structure, which shows that the side chains of R3, S8 and W10 are directly involved in BC2-Nb interactions while permutants of A6 are probably sterically disfavored and may prevent the formation of the headlock binding. Taken together, our analysis confirms that a small subset of side-chain interactions helps determine the specificity of BC2-Nb for the BC2T sequence.

### Generation of a BC2T-based capture system

**Figure 3:**
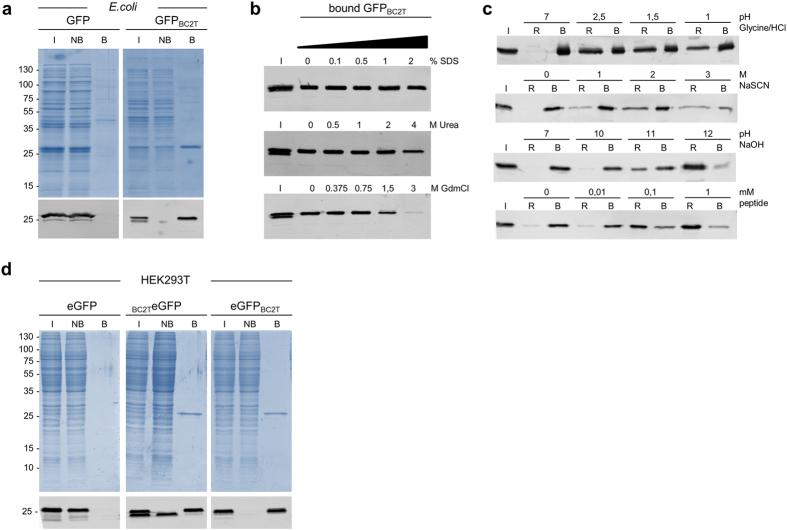
One-step purification of BC2-tagged proteins using the BC2 nanotrap. (**a**) For immunoprecipitation soluble protein fractions of bacterial lysates either expressing GFP with a C-terminal BC2 tag (GFP_BC2T_) or solely GFP were incubated with the BC2-Nb immobilized on agarose (BC2 nanotrap). Input (I), non-bound (NB) and bound fractions (B) were separated by SDS-PAGE and visualized either by Coomassie Blue (top) or by immunoblot analysis (bottom). (**b**) The BC2 nanotrap efficiently binds its epitope under harsh conditions. GFP_BC2T_ derived from bacterial extracts was incubated at increasing levels of SDS (0–2%), Urea (0–4 M) or guanidinium hydrochloride (0–3 M) and precipitated as described. Shown are the Input (I) and bound fractions at indicated conditions. (**c**) BC2-tagged proteins are efficiently released using alkaline pH or peptide elution. GFP_BC2T_ bound to the BC2 nanotrap was subjected either to elution with sodium thiocyanate (NaSCN, 1–3 M), acidic elution (0.2 M glycine pH 1–2.5), alkaline elution (pH 10–12) or peptide elution (0–1 mM). Aliquots of released (R) and bound (B) fractions were analyzed by immunoblotting with an anti-GFP antibody. (**d**) The BC2 nanotrap bind BC2-tagged proteins from human cell lysates irrespectively whether the BC2 tag is located on the N- or the C-terminus. For immunoprecipiation of BC2-tagged proteins soluble protein fractions of HEK293T cells expressing _BC2T_ eGFP, eGFP_BC2T_ or solely eGFP were incubated with the BC2 nanotrap as described. Input (I), non-bound (NB) and bound fractions (B) were separated by SDS-PAGE and visualized either by Coomassie Blue staining (top) or immunoblot analysis (bottom).

Based on the unusual ligand binding mode of the BC2-Nb we aimed to develop a BC2T-based affinity system. We covalently coupled purified BC2-Nb to Sepharose beads and generated an affinity matrix that we refer to as BC2 nanotrap. First, we tested the ability of the BC2 nanotrap to precipitate BC2T-tagged proteins directly from crude lysates. Therefore, the soluble protein fractions of *E. coli* cells expressing either C-terminally tagged GFP (GFP_BC2T_) or wtGFP (control) were incubated with the BC2 nanotrap and the input, non-bound and bound fractions were analyzed by SDS-PAGE followed by coomassie staining and immunoblotting (Fig. 3a). Our data shows that the BC2 nanotrap quantitatively precipitates GFP_BC2T_. Next, we performed BC2T purification in the presence of various non-denaturing detergents (NP-40, Triton X100, CHAPS or Tween 20, 0.1–1% w/v) or increasing salt concentrations (0–500 mM NaCl, 2–50 mM KCl, 2–20 mM MgCl_2_). None of these reagents appeared to have an impact on binding efficiency (data not shown). Additionally, we tested antigen binding under denaturing conditions by raising the concentrations of sodium dodecyl sulfate (SDS) or chaotropic agents (GdmCl; Urea) in the binding buffer. We observed that the BC2 nanotrap efficiently precipitates its antigen in the presence of 2% SDS, 4 M Urea or up to 1.5 M GdmCl (Fig. 3b). This indicates that the BC2 nanotrap remains functionally active under harsh conditions.

Although in some cases such harsh binding and elution conditions might be favorable to obtain highly pure protein, most of the bound protein is presumably denatured and does not maintain biological activity. Hence, we tested more gentle elution conditions using MgCl_2_ (0.5 M–4 M), sodium thiocyanate (NaSCN, 1–3 M) or pH-mediated release (acidic; pH 1–2.5 or alkaline; pH 10–12). We also tested liberation of bound GFP_BC2T_ by competitive elution adding increasing concentrations of BC2 peptide (PDRKAAVSHWQQ, 0.01–1 mM). Incubation with MgCl_2_ does not elute GFP_BC2T_ (data not shown), whereas treatment with high concentrations of NaSCN or acidic elution (pH 1.5) resulted in the release of 30–40% of bound protein (Fig. 3c, upper panels). In contrast, alkaline elution using higher pH (pH 11 and 12) revealed a more efficient release of 40–80%. Notably, competitive elution was highly efficient as ~60% and ~80% of GFP_BC2T_ were detected in elution fractions after addition of 0.1 mM and 1 mM BC2 peptide, respectively (Fig. 3c, lower panel). Moreover, whereas the fluorescence of GFP was drastically affected upon treatment with NaSCN or acidic pH, alkaline pH or peptide elution yielded fully fluorescent GFP (Supplementary Fig. 4). These results show that the BC2 peptide can efficiently displace BC2-bound proteins in their natively folded state.

We further analyzed the BC2-capture system for one-step purification of recombinant proteins derived from human cells. Specifically, we investigated whether the terminal position of the BC2 tag has an impact on binding. To this end, we expressed a modified GFP comprising the BC2 tag either on the N- (_BC2T_ eGFP) or the C-terminus (eGFP_BC2T_) in human embryonic kidney (HEK) 293T cells using untagged eGFP as a negative control. Two days after transfection we generated soluble protein fractions and subjected them to immunoprecipitation using the BC2 nanotrap. Input, non-bound and bound fractions were analyzed by SDS-PAGE followed by coomassie staining and immunoblotting (Fig. 3d). The results show that both N- and C-terminally BC2-tagged GFP constructs were efficiently precipitated by the BC2 nanotrap whereas no GFP was detected in the negative control. A slightly smaller GFP fragment appeared in the non-bound fraction of _BC2T_ eGFP. Since this band does not appear in the bound lane we hypothesized that it appears due to protease-mediated removal of the N-terminal BC2 tag during the cellular lysis procedure.

Next, we tested whether the BC2 nanotrap can precipitate BC2-tagged cellular components of the intermediate filaments or the nuclear replication machinery. We genetically fused the BC2T either to mCherry-vimentin (mCherry-VIM_BC2T_) or eGFP-PCNA (eGFP-PCNA_BC2T_) and expressed both constructs in HEK293T cells. As controls, we used corresponding constructs without the BC2 tag. Soluble protein extracts were incubated with the BC2 nanotrap and whole lysate (input), non-bound and bound fractions were analyzed by immunoblotting using anti-vimentin or anti-PCNA antibodies. The results show that both mCherry-VIM_BC2T_ and eGFP-PCNA_BC2T_ were efficiently precipitated with the BC2 nanotrap, while no signal was detectable in the bound fractions of the untagged proteins (Supplementary Fig. 5a). The next question was whether the BC2-Nb can also detect BC2-tagged proteins in immunoblotting. Hence, we generated fluorescently labeled BC2-Nb by chemically coupling the purified nanobody to the organic dye AlexaFluor488 (BC2-Nb_AF488_) and used it to probe immunoblots with soluble protein fractions of cells expressing eGFP-PCNA, eGFP-PCNA_BC2T_, mCherry-VIM or mCherry-VIM_BC2T_. The results show that BC2-Nb_AF488_ is highly specific for BC2-tagged proteins, whereas untagged proteins were not detected (Supplementary Fig. 5b). Next, we asked whether the BC2-Nb also recognizes endogenous β-catenin in the presence of BC2-tagged proteins. While no signal for β-catenin was detected in Western blot with the BC2-Nb_AF488_ (Supplementary Fig. 5b) minor amounts of β-catenin compared to the overexpressed BC2-tagged proteins were found in the bound fractions after precipitation with the BC2 nanotrap (Supplementary Fig. 5c). Finally, we analysed whether the BC2 tag has an impact on protein expression or solubility. Therefore we compared the amounts of GFP-PCNA, GFP-PCNA_BC2T_, mCherry-VIM and mCherry-VIM_BC2T_ expressed in mammalian cells. Data derived from immunoblotting revealed comparable expression levels of the corresponding proteins in the soluble fractions (Supplementary Fig. 6a). Furthermore, live cell imaging showed that the overall expression levels, characteristic localization or solubility of the tested proteins are not affected by the addition of the BC2 tag (Supplementary Fig. 6b).

In summary, the results show that the BC2T/BC2-Nb affinity system enables a robust and convenient one-step purification of recombinant proteins from different expression systems under native and denaturing conditions. In combination with the observed functionality in immunoblot detection, the system covers the full range of capture and detection of BC2-tagged proteins in biochemical analysis.

### Immunocytochemistry using fluorescently labeled BC2-Nb

**Figure 4:**
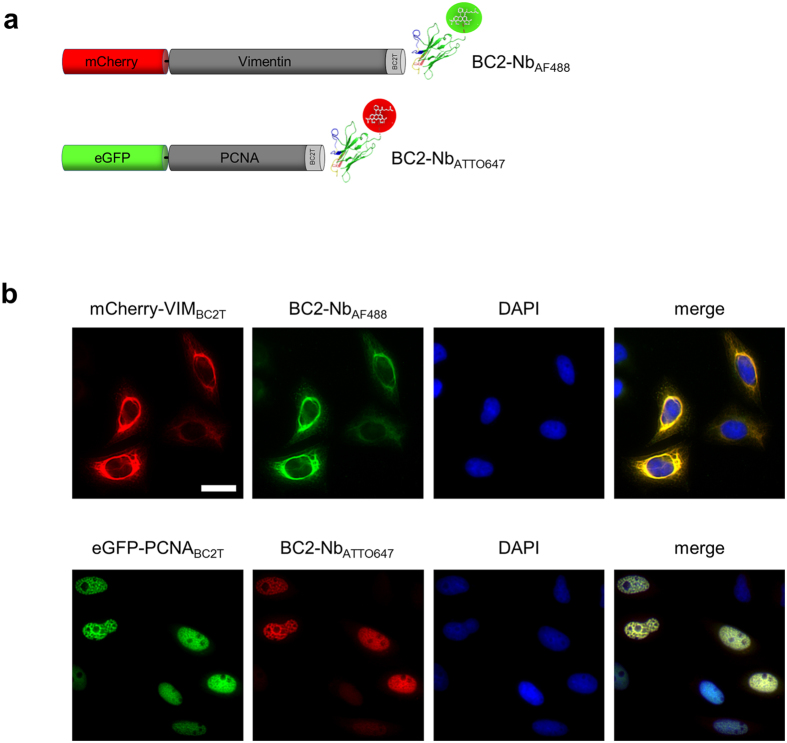
Immunocytochemistry using fluorescently labeled BC2 nanobody. (**a**) Schematic representation of BC2-tagged fusion proteins and fluorescently labeled BC2-Nbs used for co-localization studies. The BC2T sequence was genetically fused to the C-terminus of mCherry-Vimentin (mCherry-VIM_BC2T_) and eGFP-PCNA (eGFP-PCNA_BC2T_). (**b**) HeLa cells ectopically expressing mCherry-VIM_BC2T_ or eGFP-PCNA_BC2T_ were fixed either with methanol or PFA, respectively, followed by staining with the indicated fluorescently labeled BC2-Nbs and DAPI. Scale bar 25 μm.

Numerous nanobodies have been described for molecular imaging of disease-relevant antigens located on cellular surfaces^20^,^21^,^22^. There are very few studies so far which have used nanobodies for cellular imaging^23^. We therefore tested whether the fluorescently labeled BC2-Nb (coupled to the organic dyes AlexaFluor488 and ATTO647 (BC2-Nb_AF488_; BC2-Nb_ATTO647_)) is suitable to visualize BC2-tagged cellular proteins. To generate relevant cellular target structures we expressed the previously described fusion constructs mCherry-VIM_BC2T_ or GFP-PCNA_BC2T_ in human cells (Fig. 4a). Fluorescent constructs of vimentin become incorporated into the cellular intermediate filament network and visualize vimentin fibers in the cytoplasm upon transient cellular expression^24^, whereas GFP-PCNA is found at sites of DNA replication forming characteristic spot-like structures in the nucleus during the S phase of the cell cycle^25^. The characteristic pattern of the applied fusion proteins enables us to perform co-localization studies using immunocytochemistry with the dye-labeled BC2-Nbs.

By staining of HeLa cells expressing mCherry-VIM_BC2T_ with the BC2-Nb_AF488_ we observed a strong co-localization of the green nanobody signal along cytoplasmic vimentin structures shown in the red channel (Fig. 4b, Supplementary Fig. 7a). Correspondingly, the BC2-Nb_ATTO647_ revealed a clear co-localization with GFP-PCNA_BC2T_ at replication foci during the S phase. The signals of both, GFP-PCNA_BC2T_ and BC2-Nb_ATTO647_ were exclusively found in the nucleus, demonstrating the binding specificity of BC2-Nb. No nanobody signal was detected in cells expressing mCherry-VIM or GFP-PCNA constructs lacking the BC2 tag (Supplementary Fig. 7b). These data demonstrate that the fluorescently labeled BC2-Nb specifically binds to ectopically expressed BC2T fusion proteins and can therefore be applied for direct antigen detection in immunocytochemistry.

## Discussion

Nanobodies are emerging cell-biological tools that have proven to be invaluable for applications ranging from co-crystallization studies to live-cell imaging^12^,^26^,^27^. They combine the outstanding potential of *in vivo* matured antibodies with recombinant protein technologies^28^. Most currently available nanobodies recognize conformational epitopes rather than linear peptides^6^. This observation might be due to the fact that peptide-binding nanobodies have lower affinities caused by an insufficient binding interface and, consequently, are not enriched during recombinant selection processes such as phage display. However, as an exception to the rule very few nanobodies have been identified so far that bind linear peptides with affinities in the nanomolar range^29^,^30^.

We have generated a nanobody that binds a short, linear peptide with very high affinity (K_D_ ~1.4 nM). Our structure analysis of the BC2-Nb/BC2-peptide complex revealed an unusual binding mode in which the BC2 peptide adopts a β-strand conformation that is part of a β-sheet structure formed by the CDR3 and the framework regions. Numerous backbone hydrogen bonds provide affinity, and a salt bridge between Arg106 of CDR3 and Glu44 of FR2 embraces the bound peptide in a headlock-like fashion. A comparison with all nanobody complexes available in the Protein Data Bank (PDB) shows that such a binding mode has not been previously observed in any of these structures. Affinity measurements of headlock-mutated BC2-Nbs show ~10-fold reduced binding affinities and higher off-rates. These data clearly suggest that the headlock helps to fasten the already-bound peptide to the nanobody.

Epitope analysis using positional scanning peptide libraries revealed a small set of four specificity-determining residues, of which W10 is the most critical. By contrast, the remaining eight residues do not contribute towards the binding specificity, mostly because their side chains do not engage in contacts. The reliance on many backbone interactions to generate affinity and only four amino acid side chains for specificity renders the BC2T/BC2-Nb system especially versatile. Most of the BC2T residues can be replaced without sacrificing binding efficiency, and this allows a straightforward adaptation of the tag for individual applications, including charge modifications, addition of tryptophan residues for increased absorbance at 280 nm, introduction of a protease cleavage site or even an unusual amino acid for labeling purposes.

Epitope tagging is a standard method that enables rapid and effective purification and *in vivo* localization of recombinant proteins. Since large protein tags including GST, MBP or GFP sometimes sterically interfere with subcellular protein localization or folding, small peptide-based epitope tags are preferable^31^. Such tags which have either a synthetic origin (FLAG) or are derived from viral (HA, V5) or endogenous mammalian (c-myc, CBP) proteins, are characterized by a size of 8–26 amino acid residues and are detected by classical IgGs (poly- or monoclonal)^32^. Although many immunoaffinity capture systems are available using tag-specific antibodies, there are still severe problems due to low affinity binding, unspecific interactions, batch to batch variations or reduced functionality of the antibodies upon covalent coupling to solid surfaces.

The BC2-based capture and detection system described in this study is an attractive alternative to currently available epitope tag systems. We have shown that the BC2-Nb binds BC2-epitope-tagged proteins with a K_D_ of ~1.4 nM and therefore shows a ~10–100-fold higher affinity compared to available FLAG-, HA-, c-myc- or the nanobody-derived EPEA-systems^29^,^33^,^34^. The latter has been shown to be functional in capturing recombinant proteins comprising the amino acid sequence E-P-E-A, but only when it is placed at the C-terminus^35^. By covalently coupling the monovalent BC2-Nb to solid matrices we generated the BC2 nanotrap. This serves as a highly efficient pulldown reagent similar to the previously described GFP nanotrap^11^ which is widely used to purify GFP-tagged complexes from cellular lysates^6^. The excellent binding capacities of the BC2 nanotrap are favorable for proteomic analysis applying e.g. highly competitive binding conditions using chaotropic agents (up to 4 M Urea or 1.5 M GdmCl) or denaturing detergents (2% SDS) in the binding reaction^36^. In contrast to the GFP nanotrap, where bound proteins are released only under denaturing conditions, BC2 nanotrap-bound proteins can be eluted in a functional conformation simply by peptide competition using low amounts of BC2-peptide. Positional cloning revealed that the N- or the C-terminally localized BC2 tag is equally well recognized by the BC2 nanotrap. For N-terminally BC2-tagged GFP we observed an additional band in the non-bound fraction. This indicates that the BC2 tag might be proteolytically removed upon cellular lysis, a phenomenon which has already been described for other epitope tags in mammalian expression systems^37^. In addition to its capacity to precipitate BC2-tagged proteins, the fluorescently modified BC2-Nb directly detects BC2-tagged proteins in Western blots without the need of a secondary antibody. The different functional embodiments show that the BC2-Nb is a versatile and robust tool for numerous biochemical studies.

Finally, our study shows that the BC2-Nb offers new opportunities for cellular imaging. Basically nanobodies can be easily modified by site-directed labelling with the full-range of available organic dyes^23^(Romer *et al.*, unpublished data) and applied for direct imaging of cellular structures^23^,^38^ without the need of a secondary antibody for detection. Recently, it was demonstrated that GFP- and RFP-specific nanobodies labeled with photoactivated localization microscopy (PALM)-compatible organic dyes such as AlexaFluor 647 (AF647) efficiently visualize fluorescent fusion proteins in high-resolution microscopy^23^. There is an ongoing demand for nanobodies that recognize a smaller epitope to minimize steric hindrances or potential linkage errors derived from large tags such as GFP- or RFP. In this study we demonstrate for the first time the visualization of distinct subcellular structures such as the intermediate filamentous network or an essential part of the replication machinery using a fluorescently labeled peptide-specific nanobody. Based on our findings we propose that a BC2-mediated detection system provides unique advantages compared to currently available approaches. Firstly, due to its small size the BC2 tag does not interfere with the subcellular localization or folding of ectopically expressed proteins. Secondly, the usage of a small nanobody (~2 × 4 nm) directly links the detection signal to the corresponding antigen. Thirdly, the BC2 nanobody can be easily modified e.g. for site-directed labeling with appropriate high resolution compatible dyes. In summary, nanobody-mediated labeling of BC2-tagged constructs now combine a minimal epitope tag with the high photon yield of organic dyes and minimal linkage error.

## Methods

### Plasmids and protein expression/purification

Detailed description of all expression constructs as well as protein expression and purification is provided in the Supplementary methods section.

### Complex formation

For complex formation, a BC2-Nb solution at 2 mg/ml was mixed and incubated with a threefold molar excess of peptide in 10 mM Tris/HCl pH 7.4, 100 mM NaCl buffer for 1 h at room temperature. Excess of peptide was removed via size exclusion chromatography (Superdex 200 increase, GE Healthcare). Complex formation was confirmed by liquid chromatography mass spectrometry using a Shimadzu LCMS 2020 with a Phenomenex Kinetex (2.6 u C18 100 Å) column. The complex was concentrated to 13.7 mg/ml and used for crystallization experiments.

### Crystallization

Unliganded BC2-Nb was crystallized using hanging drop vapor diffusion by mixing 3.2 mg/ml protein solution with crystallization buffer (0.1 M MES/imidazole pH 6.7, 12.5% [v/v] 2-Methyl-2,4-pentanediol (MPD), 7.5% [w/v] PEG 1000, 7.5% [w/v] PEG 3350, 3 mM alcohol mix (Molecular Dimensions)) in a 1:1 ratio at 20 °C. The BC2-Nb/BC2T complex was crystallized using hanging drop vapor diffusion, mixing 13.7 mg/ml complex solution with crystallization buffer (0.1 M MES/imidazole pH 6.5, 12.5% [v/v] 2-Methyl-2,4-pentanediol (MPD), 12.5% [w/v] PEG 1000, 12.5% [w/v] PEG 3350, 4 mM amino acid mix (Molecular Dimensions)) in a 1:1 ratio at 20 °C. In both cases, crystals were transferred into crystallization solution containing 30% [v/v] MPD for cryoprotection, and flash cooled in liquid nitrogen after incubation for 30 s. One Dataset from BC2-Nb and four dataset from the same crystal of BC2-Nb/BC2T complex at different positions were collected with a beam wavelength of 0.918409 Å at beamline MX 14.2 of BESSY II at the Helmholtz-Zentrum Berlin (HZB).

X-ray data were reduced and, in the case of BC2-Nb/BC2T complex, merged using the XDS package^39^. Initial phases for the BC2-Nb data were obtained by molecular replacement using PHASER^40^ with a CHAINSAW^41^,^42^ modified model of a nanobody (PDB ID: 2×1O) containing only the core region. The structure of the BC2-Nb/BC2T complex was then solved using the unliganded BC2-Nb structure as a search model in molecular replacement. Both structures were refined using PHENIX.refine^43^, REFMAC5^44^ and COOT^45^. The two structures were validated with MOLPROBITY^46^. The Ramachandran plot shows 100% (BC2-Nb), 98.1% (BC2-Nb/BC2T complex) in favored and 100% in allowed regions.

### Structure visualization and analyzation

Superpositions of structures and calculation of RMSD values were conducted using the Phenix structure_comparison. Images of crystal structures were prepared with PyMol (http://www.pymol.org).

### Mass spectrometry analysis

Detailed description of mass spectrometry analysis of binding specificities with synthetic positional scanning peptide libraries is provided in the Supplementary methods section.

### Immunoprecipitation of BC2-tagged proteins

HEK293T cells transiently expressing eGFP, eGFP_BC2T_ or _BC2T_ eGFP, eGFP-PCNA, eGFP-PCNA_BC2T_, mCherry-Vimentin, mCherry-Vimentin_BC2T_ were washed and harvested in phosphate buffered saline (PBS), snap-frozen in liquid nitrogen and stored at −20 °C. Cell pellets were homogenized in 200 μl lysis buffer (10 mM Tris/Cl pH 7.5, 150 mM NaCl, 0.5% NP40, 1 μg DNaseI, 2 mM MgCl_2_, 2 mM PMSF, 1 × protease inhibitor mix M (Serva) by repeated pipetting for 40 min on ice. After a centrifugation step (10 min at 18.000 × g) the protein concentration of each lysate was determined using a Pierce BCA Protein Assay Kit (Thermo Fisher Scientific) according to manufacturer’s protocol and the protein solutions were adjusted with dilution buffer (10 mM Tris/Cl pH 7.5, 150 mM NaCl, 2 mM PMSF) to a concentration of 1 mg/ml. 2% of the supernatant were added to SDS-containing sample buffer (referred to as input). 50 μl BC2 nanotrap (slurry) per 100 μl supernatant were added followed by incubation for 1 h on an end-over-end rotor at 4 °C. After a centrifugation step (2 min, 2.500 × g) the precleared supernatant was transferred to a new cup. 2% were added to SDS-containing sample buffer (referred to as non-bound). After four washing steps, BC2 nanotrap bound proteins were eluted by boiling the beads in 50 μl SDS-sample buffer or by incubation with indicated elution buffers. Samples were analyzed by SDS-PAGE followed by immunoblotting. Immunoblots were probed with indicated antibodies. Detailed description of immunoprecipitation at harsh conditions or elution of bound BC2-tagged proteins is provided in the Supplementary methods section.

### Immunofluorescent staining with fluorescently labeled nanobody

Purified BC2 nanobody (1 mg) was labeled with the NHS-activated fluorescent dyes Alexa488 (Life technologies) or Atto647 (Atto-Tec) according to manufacturer’s guidelines. After coupling, unbound dye was removed by separation on PD-10 Desalting Columns (GE Healthcare). For immunoblot analysis, 5 μg of BC2-Nb_AF488_ was diluted in 4 ml 3% BSA diluted in TBS, 0.05% Tween20 and Western blots were incubated for 1 h at RT.

For immunocytochemistry 5–10 × 10^3^ adherent HeLa cells per well of a μClear 96 well plate (Greiner) were transfected with plasmids coding for C-terminally BC2-tagged vimentin or PCNA. After 24 h, cells were washed once with PBS and fixed with 4% w/v paraformaldehyde (PFA) in PBS for 15 min at RT or with methanol for 15 min at −20 °C. After removal of the fixative and washing twice with PBS, cells were blocked and permeabilized with 3% w/v BSA and 0,1% v/v Triton X-100 in PBS for 1 h at RT. Subsequently, labeled BC2 nanobody was added with a final concentration of 10–15 μg/ml and 4′,6-diamidino-2-phenylindole (DAPI, Sigma Aldrich) with a final concentration of 1 μg/ml for overnight incubation at 4 °C. Unbound nanobody and DAPI were removed by washing 3 times with a mixture of PBS and 6% v/v 5 M NaCl in H_2_O. Images of Fig. 4b and Supplementary Fig. 7b were acquired with an Image Xpress micro XL system. Confocal images of Supplementary Fig. 7a were acquired with an ImageXpress Micro Confocal (kindly provided by Christian Holz, Molecular Devices).

## Additional Information

**How to cite this article**: Braun, M. B. *et al.* Peptides in headlock – a novel high-affinity and versatile peptide-binding nanobody for proteomics and microscopy. *Sci. Rep.* **6**, 19211; doi: 10.1038/srep19211 (2016).

## Supplementary Material

Supplementary Information

## Figures and Tables

**Table 1:** Mutation of the “headlock” motif leads to an increased dissociation. The table summarizes affinities (K_D_), association (k_on_) and dissociation constants (k_off_) determined for BC2-Nb, BC2-Nb_R106E_ and BC2-Nb_R106S_. Corresponding sensograms are shown in Supplementary Figure 3.

Nbs	R_max_ [RU]	K_D_ [nM]	k_on_ [M^−1^s^−1^]	k_off_ [s^−1^]	Chi^2^
BC2-Nb	170 ± 0.34	1.4 ± 0.06	4.6 ± 0.04 × 10^5^	6.4 ± 0.27 × 10^**−**4^	3.5
BC2-Nb_R106E_	160 ± 0.48	12.0 ± 0.15	2.8 ± 0.01 × 10^5^	3.4 ± 0.04 × 10^**−**3^	3.8
BC2-Nb_R106S_	160 ± 0.43	9.7 ± 0.13	3.3 ± 0.03 × 10^5^	3.2 ± 0.03 × 10^**−**3^	3.1
