# Supplementary material for: Peptides in headlock – a novel high-affinity and versatile peptide-binding nanobody for proteomics and microscopy
Source: Sci Rep. 2016 Jan 21;6:19211. doi: 10.1038/srep19211 (PMC4726124; doi:10.1038/srep19211)
Supplement: Supplementary Information [file srep19211-s1.pdf]

Peptides in headlock – a novel high-affinity and versatile peptide-binding nanobody for proteomics and microscopy

Michael B. Braun[1], *Bjoern Traenkle*[2], *Philipp A. Koch*[2], *Felix Emele*[2], *Frederik Weiss*[3], *Oliver Poetz*[3], *Thilo Stehle*[1,#]* *and Ulrich Rothbauer*[2,3#]*

[1] Interfaculty Institute of Biochemistry, Eberhard-Karls University Tuebingen, Germany

[2] Pharmaceutical Biotechnology, Eberhard-Karls University Tuebingen, Germany

[3] Natural and Medical Sciences Institute at the University of Tuebingen, Reutlingen, Germany

[4] Vanderbilt University School of Medicine, Nashville, TN, USA

* both authors contributed equally to this work

[#] corresponding authors

Correspondence to:

Thilo Stehle, Interfaculty Institute of Biochemistry (IFIB), Hoppe-Seyler-Str. 4, 72076 Tuebingen, Germany

E-mail: thilo.stehle@uni-tuebingen.de

Ulrich Rothbauer, Dept. of Pharmaceutical Biotechnology, University of Tuebingen, Markwiesenstr. 55, 72770 Reutlingen, Germany.

E-mail: ulrich.rothbauer@uni-tuebingen.de

Supplementary information

Supplementary Figure 1

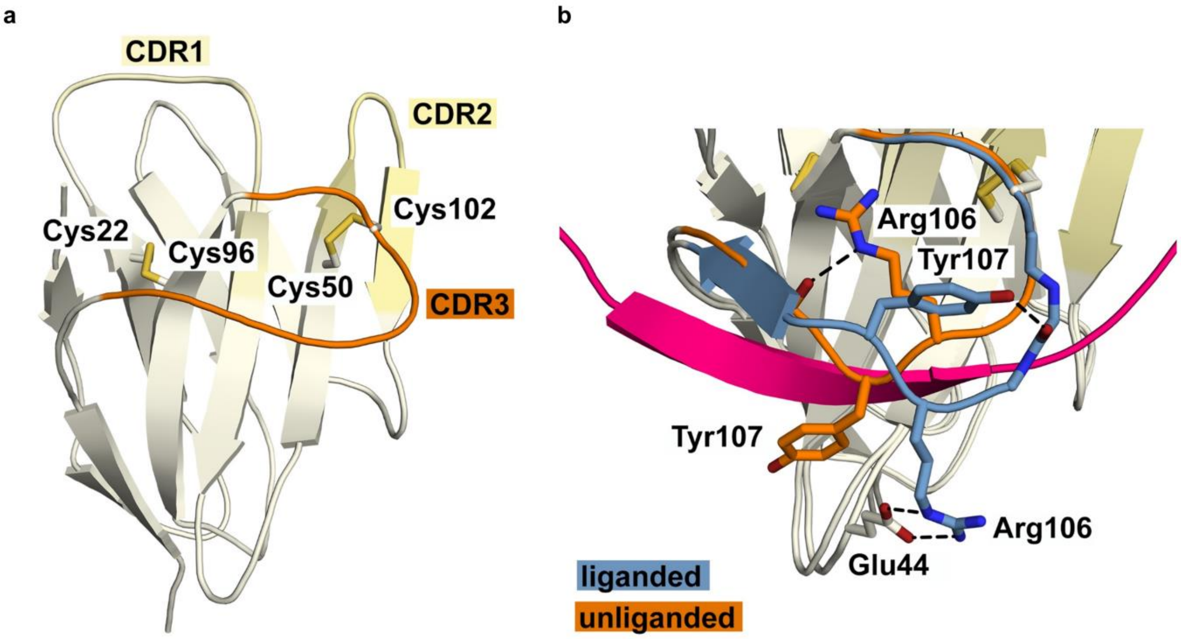

Structure of unliganded BC2-Nb and comparison with the BC2-Nb/BC2T complex.

(a) Ribbon drawing of the unliganded BC2-Nb structure. The four cysteine residues forming two disulfide bonds are marked. CDR3, which contributes contacts with the peptide in the liganded structure and undergoes an amino acid flip upon binding, is highlighted in orange. **(b)** Superposition of unliganded and liganded BC2-Nb structures. Comparison of the two structures reveals a 180° flip of two amino acids. In the BC2-Nb structure, Arg106 is interacting with the carbonyl group of Glu108 and Tyr107 is involved in a cation π-interaction with Arg45 (orange ribbon tracing). The β-carbon of Arg106 is orientated towards and Tyr107 away from the Nb. In the peptide bound complex structure it is the other way around (blue ribbon tracing). Arg106 is involved in the “headlock” binding and Tyr107 is forming a hydrogen bond to the carbonyl group of Arg104.

Supplementary Figure 2

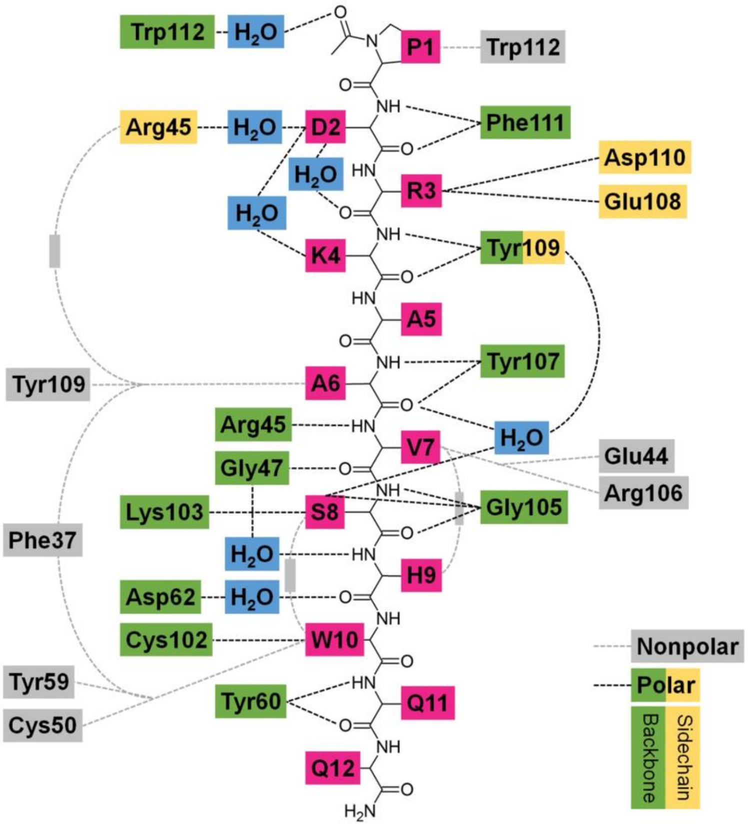

Scheme of interactions of the BC2-peptide with the BC2-Nb.

The BC2-peptide is shown with side chains in pink. Its acetylated N-terminus is at the top and the amidated C-terminus is at the bottom. All polar interactions within 3.5 Å are represented with dotted black lines, with their interaction partners from the BC2-Nb in green for backbone and yellow for side chain interactions. Waters are shown in blue. Relevant hydrophobic interactions within 4.0 Å are represented with grey dotted lines.

Supplementary Figure 3

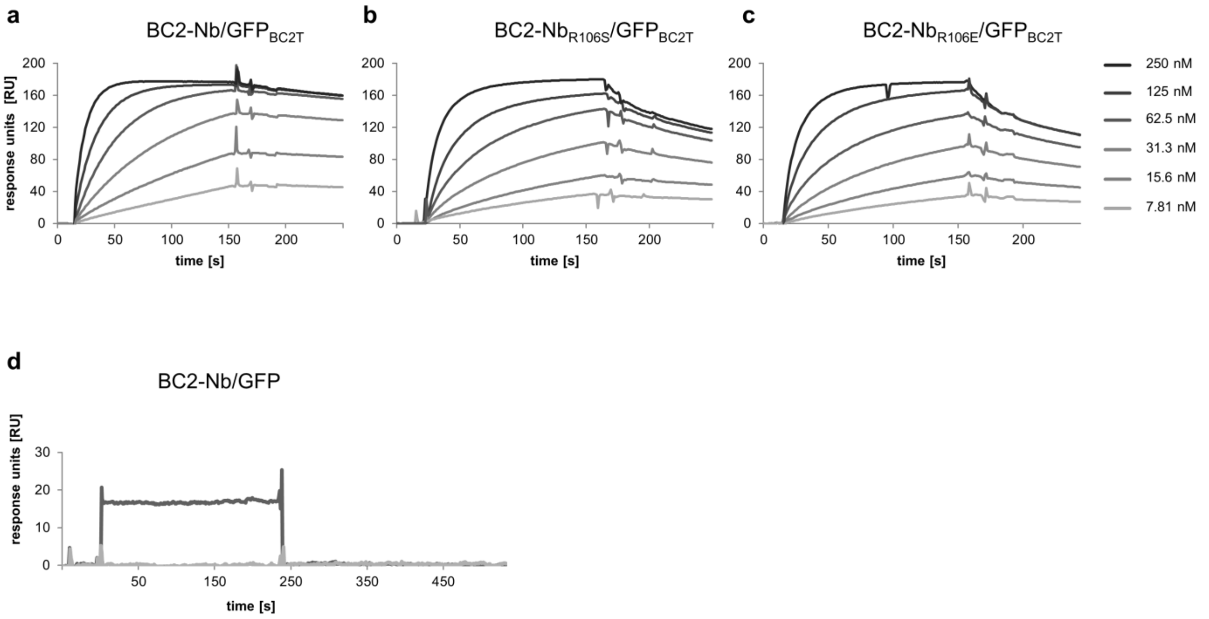

Mutation of the headlock motif leads to increased off-rates of the BC2 nanobody.

For surface plasmon resonance spectroscopy (SPR)-based affinity measurements, GFP with a C-terminal BC2-tag (GFP_{BC2T}) (**a** - **c**) or solely GFP (**d**) was immobilized on a CM5-chip. Kinetic measurements were performed by injecting six concentrations of BC2-Nb (**a**), BC2-Nb_{R106S} (**b**) or BC2-Nb_{R106E} (**c**) ranging from 8 nM – 250 nM. The obtained data sets were evaluated using the 1:1 Langmuir binding model with mass transfer. As a control BC2-Nb was tested for binding to GFP only (**d**). The obtained affinities (K_D), association (K_{on}) and dissociation constants (K_{off}) determined for BC2-Nb and the corresponding mutants are summarized in **Table 1** of the main manuscript.

Supplementary Figure 4

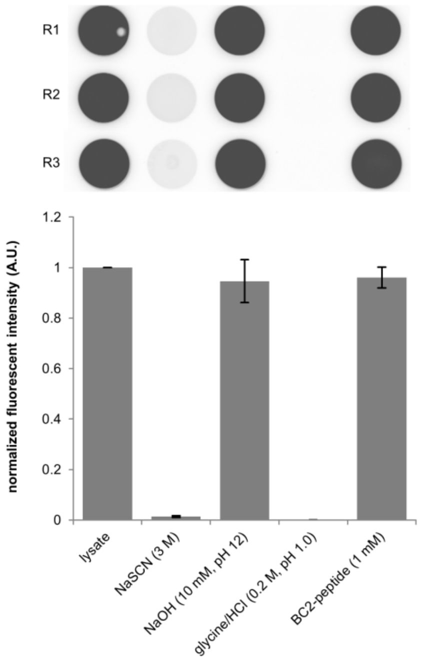

Proteins captured by the BC2 nanotrap can be eluted under native conditions.

GFP_{BC2T} derived from bacterial lysate was bound by the BC2 nanotrap and subjected to elution using buffer conditions as described in **Fig. 3 c**. The fluorescence signal intensity of released GFP_{BC2T} was visualized and quantified using a laser scanner. Upper panel shows the fluorescent intensity of eluted GFP_{BC2T} in comparison of full lysate. Lower panel: Quantification of GFP-fluorescence obtained for different elution conditions. GFP-fluorescence obtained from untreated lysate was set to 1. Means and s.d. (error bars) of three independent experiments are shown (R1 - R3).

Supplementary Figure 5

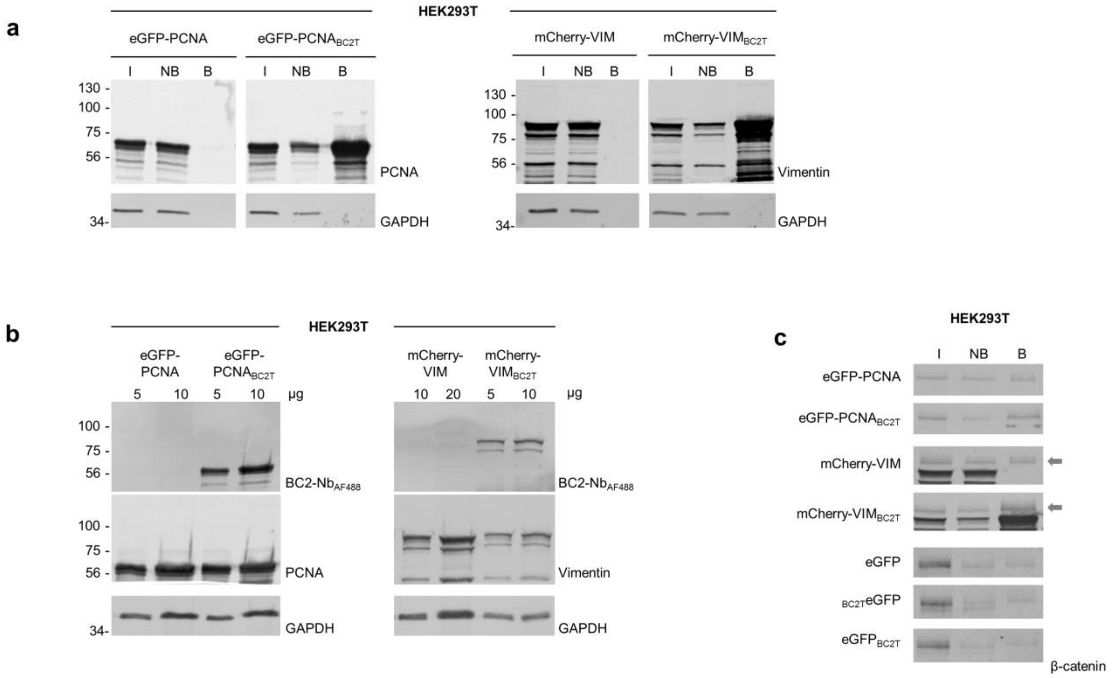

BC2-Nb is functional for immunoprecipitation and detection of BC2-tagged proteins.

(**a**) For immunoprecipitation soluble proteins fractions of HEK293T cells either expressing eGFP-PCNA (control) or BC2-tagged eGFP-PCNA (eGFP-PCNA$_{BC2T}$) (left panel) or mCherry-Vimentin (mCherry-VIM, control) or BC2-tagged mCherry-VIM (mCherry-VIM$_{BC2T}$) were incubated with the BC2 nanotrap. Input (I), non-bound (NB) and bound fractions (B) were separated by SDS-PAGE and visualized either by immunoblot analysis using anti-PCNA or anti-Vimentin antibodies (upper panel). As loading control blots were probed with an anti-GAPDH antibody (lower panel). (**b**) For Western blot detection using fluorescently labeled BC2-Nb (BC2-Nb$_{AF488}$) indicated amount of the input fractions (as shown in (**a**)) were subjected to SDS-PAGE and immunoblotting. The Western blots were probed with BC2-Nb$_{488}$ followed by detection with anti-PCNA (left panel) or anti-Vimentin (right panel) antibodies. As loading control blots were probed with an anti-GAPDH antibody (lower panel). (**c**) BC2 nanotrap precipitates only minor amounts of endogenous β-catenin compared to overexpressed BC2-tagged proteins. Samples

as described in (**A**) or **Fig. 3 d** were subjected to immunoblot analysis with an anti-β-catenin antibody. Arrows are indicating β-catenin specific signals.

Supplementary Figure 6

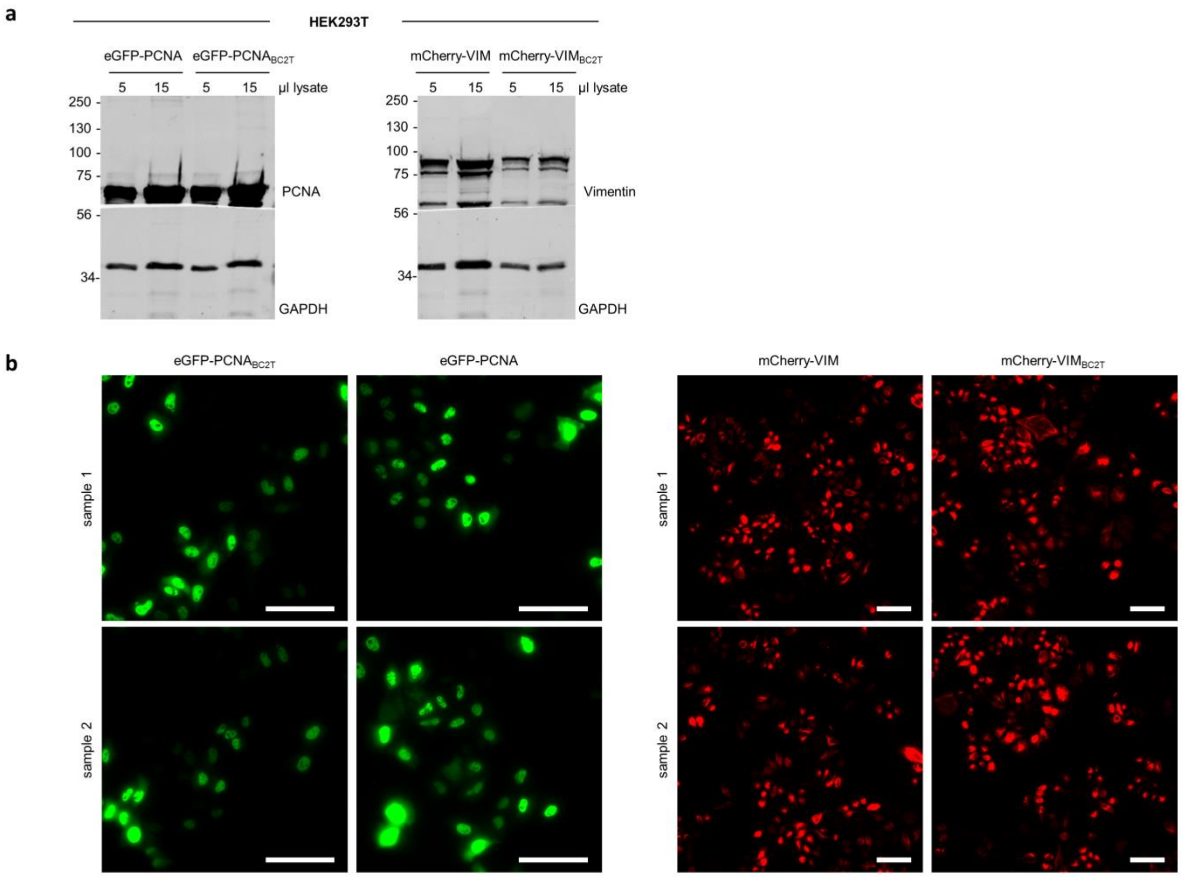

Addition of the BC2-tag has no impact on expression levels or solubility of the fusion proteins.

(**a**) Soluble protein fractions of HEK293T cells transiently expressing eGFP-PCNA or eGFP-PCNA$_{BC2T}$ (left panel) or mCherry-VIM or mCherry-VIM$_{BC2T}$ (right panel) were separated by SDS-PAGE and visualized by immunoblot analysis using anti-PCNA or anti-Vimentin antibodies. As loading control blots were probed with an anti-GAPDH antibody.

(**b**) HeLa cells were transiently transfected with expression constructs coding for eGFP-PCNA or eGFP-PCNA$_{BC2T}$ respectively (left panel) or mCherry-VIM or mCherry-VIM$_{BC2T}$ (right panel). Representative images of live cells obtained from two independent transfections are shown. Scale bar 100 µm.

Supplementary Figure 7

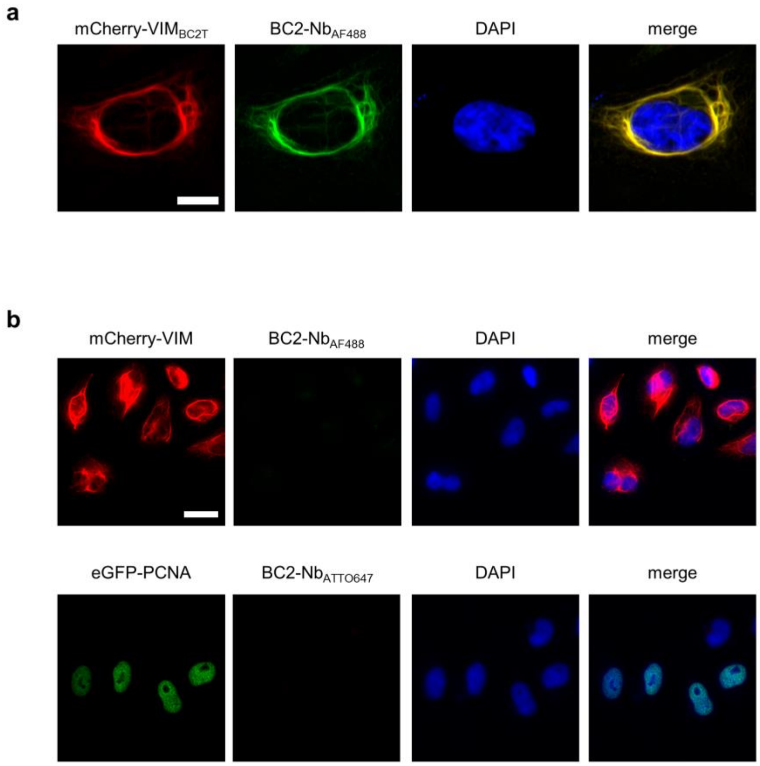

Immunocytochemistry using fluorescently labeled BC2 nanobody.

(**a**) Confocal imaging of mCherry-Vimentin$_{BC2T}$ with the BC2-Nb$_{AF488}$. HeLa cells ectopically expressing mCherry-VIM$_{BC2T}$ were fixed with methanol, followed by staining with fluorescently labeled BC2-Nb and DAPI. Shown is a maximum projection image (z-stack of 7 planes) of the cell in the lower left corner of the upper panel in **Figure 4 b**. Scale bar 10 µm. (**b**) Specificity of BC2-Nb$_{AF488}$ and BC2-Nb$_{ATTO647}$. HeLa cells ectopically expressing untagged mCherry-VIM or eGFP-PCNA were fixed with methanol or PFA, respectively, and stained with the indicated fluorescently labeled BC2-Nbs and DAPI. Scale bar 25 µm.

Supplementary Table 1

	BC2-Nb	BC2-Nb-BC2T complex*
**Data collection**		
Space group	$P2_12_12_1$ (19)	C2 (5)
Cell dimensions		
a, b, c (Å)	31.77, 47.74, 67.97	106.01, 31.53, 35.88
α, β, γ (°)	90, 90, 90	90, 107.54, 90
Resolution (Å)	39.07-1.80 (1.85-1.80)	50.55-1.00 (1.03-1.00)
R_{meas}	8.8 (94.6)	6.4 (99.7)
$I / \sigma I$	13.06 (1.45)	18.42 (2.05)
$CC_{1/2}$	99.8 (52.0)	100 (52.4)
Completeness (%)	99.6 (99.6)	99.6 (98.1)
Redundancy	14.4 (3.6)	9.5 (8.0)
Wilson B (Å^2)	27.7	11.5
**Refinement**		
Resolution (Å)	39.07-1.80	50.55-1.00
No. reflections	70062	580345
R_{work} / R_{free}	19.1 / 21.7	12.8 / 14.9
No. atoms		
BC2-Nb	902	1037
BC2T	-	115
Water	36	134
MPD	8	-
B-factors		
BC2-Nb	27.8	11.2
BC2T	-	14.3
Water	32.6	24.3
MPD	38.6	-
R.m.s. deviations		
Bond lengths (Å)	0.011	0.020
Bond angles (°)	1.107	1.452

Supplementary Table 2

This table summarizes the results of all 12 BC2T positional sequence variant libraries used in this study. For details see **Figure 2**.

BC2-Nb

	P		E		R		K		A		A		V		S		H		W		Q		Q	
	ratio	error	ratio	error	ratio	error	ratio	error	ratio	error	ratio	error	ratio	error	ratio	error	ratio	error	ratio	error	ratio	error	ratio	error
A	0.1	0.0	0.2	0.0	0.7	0.0	0.2	0.0	***0.2***	***0.0***	***0.1***	***0.0***	0.4	0.0	0.0	0.0	0.2	0.0	1.0	0.1	0.1	0.1	0.1	0.0
G	0.4	0.0	0.3	0.0	0.9	0.1	0.6	0.0	0.3	0.1	0.7	0.1	0.7	0.1	0.5	0.2	0.4	0.0	1.2	0.2	0.1	0.1	0.2	0.0
V	0.2	0.0	0.1	0.0	0.3	0.0	0.1	0.0	0.1	0.0	0.3	0.0	***0.1***	***0.0***	0.1	0.0	0.2	0.0	1.0	0.1	0.1	0.0	0.1	0.0
I/L 1	0.4	0.1	0.1	0.0	0.5	0.0	0.1	0.0	0.1	0.0	0.7	0.0	0.1	0.0	0.4	0.1	0.3	0.0	1.0	0.1	0.1	0.0	0.1	0.0
I/L 2	0.3	0.1	0.2	0.0	0.5	0.0	0.2	0.0	0.1	0.0	0.9	0.0	0.2	0.0	0.7	0.2	0.1	0.0	1.0	0.0	0.1	0.0	0.1	0.0
M	0.2	0.0	0.1	0.0	0.7	0.0	0.1	0.0	0.1	0.0	1.0	0.1	0.3	0.0	0.6	0.2	0.1	0.0	1.0	0.1	0.1	0.0	0.1	0.0
P	***0.1***	***0.0***	0.6	0.1	0.8	0.1	1.0	0.1	0.1	0.0	1.1	0.1	1.0	0.1	0.7	0.2	0.8	0.1	1.1	0.1	0.6	0.4	0.1	0.0
F	0.2	0.0	0.2	0.0	0.8	0.1	0.3	0.0	0.1	0.0	1.0	0.1	0.2	0.0	0.7	0.2	0.1	0.0	0.9	0.0	0.0	0.0	0.2	0.0
W	0.1	0.0	0.2	0.0	0.8	0.1	0.3	0.0	0.0	0.0	1.1	0.1	0.1	0.0	0.6	0.3	0.1	0.0	***0.1***	***0.0***	0.0	0.0	0.1	0.0
S	0.3	0.0	0.1	0.0	0.6	0.0	0.3	0.0	0.1	0.0	0.5	0.0	0.3	0.0	***0.1***	***0.0***	0.2	0.0	1.1	0.2	0.1	0.0	0.1	0.0
T	0.5	0.1	0.1	0.0	0.3	0.0	0.2	0.0	0.1	0.0	0.2	0.0	0.2	0.0	0.1	0.0	0.2	0.0	1.0	0.1	0.1	0.0	0.1	0.0
N	0.6	0.1	0.2	0.0	0.7	0.0	0.5	0.0	0.3	0.1	1.0	0.0	0.2	0.0	0.2	0.1	0.1	0.0	1.0	0.1	0.1	0.1	0.1	0.0
Q	0.6	0.0	0.1	0.0	0.5	0.0	0.1	0.0	0.2	0.0	1.0	0.0	0.4	0.0	0.7	0.2	0.2	0.0	1.1	0.1	***0.1***	***0.1***	***0.1***	***0.0***
Y	0.1	0.0	0.1	0.0	0.7	0.1	0.2	0.0	0.1	0.0	1.0	0.0	0.1	0.0	0.6	0.2	0.1	0.0	1.0	0.1	0.0	0.0	0.1	0.0
C (Abu)	0.1	0.0	0.1	0.0	0.5	0.0	0.1	0.0	0.1	0.0	0.1	0.0	0.2	0.0	0.0	0.0	0.2	0.0	1.0	0.0	0.1	0.1	0.1	0.0
K	0.3	0.0	0.2	0.0	0.2	0.0	***0.1***	***0.0***	0.2	0.1	1.0	0.1	0.3	0.0	0.8	0.2	0.2	0.0	1.0	0.1	0.0	0.0	0.0	0.0
R	0.2	0.0	0.1	0.0	***0.1***	***0.0***	0.1	0.0	0.2	0.2	1.1	0.1	0.2	0.0	0.5	0.3	0.1	0.0	1.0	0.1	0.0	0.0	0.1	0.0
H	0.3	0.1	0.1	0.0	0.3	0.0	0.1	0.0	0.2	0.0	1.1	0.1	0.3	0.0	0.7	0.2	***0.1***	***0.0***	1.0	0.1	0.1	0.0	0.1	0.0
D	1.0	0.1	0.1	0.0	1.0	0.1	1.0	0.0	0.5	0.1	1.0	0.1	0.5	0.0	0.8	0.2	0.1	0.0	1.0	0.1	0.2	0.1	0.6	0.1
E	0.9	0.1	***0.2***	***0.0***	1.0	0.1	0.5	0.0	0.2	0.0	1.1	0.1	0.4	0.0	0.7	0.2	0.2	0.0	1.1	0.2	0.1	0.1	0.4	0.1

BC2-NbR106S

	P		E		R		K		A		A		V		S		H		W		Q		Q	
	Ratio	error	Ratio	error	Ratio	error	Ratio	error	Ratio	error	Ratio	error	Ratio	error	Ratio	error	Ratio	error	Ratio	error	Ratio	error	Ratio	error
A	0.3	0.0	0.4	0.1	0.9	0.1	0.6	0.0	***0.4***	***0.0***	***0.2***	***0.0***	0.7	0.0	0.1	0.0	0.5	0.0	1.0	0.1	0.4	0.1	0.3	0.1
G	0.6	0.1	0.5	0.1	1.0	0.1	0.9	0.1	0.5	0.0	0.9	0.1	0.8	0.0	0.9	0.1	0.8	0.0	1.1	0.2	0.5	0.0	0.4	0.1
V	0.4	0.0	0.2	0.0	0.7	0.1	0.2	0.0	0.2	0.0	0.6	0.0	***0.3***	***0.0***	0.3	0.0	0.6	0.0	1.0	0.1	0.2	0.0	0.2	0.1
I/L 1	0.7	0.1	0.2	0.0	0.9	0.1	0.4	0.1	0.2	0.0	0.8	0.1	0.3	0.0	0.6	0.0	0.5	0.0	1.1	0.1	0.2	0.0	0.2	0.1
I/L 2	0.6	0.4	0.3	0.0	0.9	0.1	0.6	0.1	0.2	0.0	0.9	0.1	0.4	0.0	0.9	0.1	0.3	0.0	1.1	0.1	0.4	0.0	0.2	0.1
M	0.5	0.1	0.2	0.0	0.9	0.1	0.5	0.0	0.2	0.0	0.9	0.1	0.5	0.1	1.0	0.1	0.3	0.0	1.1	0.1	0.3	0.0	0.3	0.1
P	***0.2***	***0.0***	0.8	0.1	1.0	0.1	1.0	0.1	0.4	0.0	1.0	0.1	1.0	0.1	1.0	0.1	1.0	0.0	1.1	0.1	0.9	0.1	0.2	0.1
F	0.3	0.1	0.3	0.1	1.0	0.1	0.7	0.1	0.2	0.0	0.9	0.1	0.4	0.0	0.9	0.1	0.3	0.0	1.1	0.1	0.2	0.0	0.4	0.1
W	0.3	0.0	0.3	0.1	1.0	0.1	0.8	0.1	0.1	0.0	1.0	0.1	0.3	0.0	0.9	0.1	0.1	0.0	***0.1***	***0.0***	0.1	0.0	0.3	0.1
S	0.6	0.1	0.3	0.1	0.9	0.1	0.7	0.0	0.4	0.0	0.8	0.0	0.4	0.0	***0.2***	***0.0***	0.5	0.0	1.1	0.2	0.2	0.0	0.2	0.1
T	0.7	0.1	0.2	0.1	0.8	0.1	0.7	0.0	0.3	0.0	0.4	0.1	0.3	0.0	0.2	0.0	0.5	0.0	1.0	0.0	0.2	0.0	0.3	0.1
N	0.7	0.1	0.3	0.1	1.0	0.0	0.9	0.1	0.5	0.0	1.0	0.1	0.4	0.0	0.7	0.1	0.3	0.0	1.1	0.1	0.3	0.0	0.2	0.1
Q	0.7	0.1	0.2	0.0	0.9	0.1	0.4	0.0	0.3	0.0	0.9	0.1	0.5	0.0	0.9	0.1	0.4	0.0	1.1	0.2	***0.3***	***0.0***	***0.3***	***0.1***
Y	0.3	0.0	0.2	0.0	1.0	0.1	0.5	0.0	0.2	0.0	0.9	0.1	0.3	0.0	0.9	0.0	0.2	0.0	1.0	0.0	0.1	0.0	0.3	0.1
C*	0.3	0.0	0.3	0.1	0.9	0.2	0.3	0.0	0.3	0.0	0.3	0.0	0.5	0.1	0.1	0.0	0.5	0.0	1.0	0.1	0.3	0.0	0.2	0.1
K	0.5	0.1	0.2	0.1	0.5	0.1	***0.2***	***0.0***	0.2	0.0	0.8	0.1	0.3	0.0	0.9	0.1	0.3	0.0	1.1	0.1	0.1	0.0	0.1	0.0
R	0.4	0.1	0.2	0.0	***0.2***	***0.1***	0.1	0.0	0.1	0.0	0.7	0.1	0.2	0.0	0.9	0.1	0.2	0.0	1.1	0.2	0.1	0.0	0.1	0.0
H	0.6	0.1	0.2	0.0	0.7	0.1	0.3	0.0	0.3	0.0	0.9	0.1	0.4	0.0	0.9	0.1	***0.3***	***0.0***	1.1	0.1	0.2	0.0	0.3	0.1
D	0.8	0.1	0.3	0.1	1.0	0.0	1.0	0.0	0.9	0.1	1.0	0.1	0.9	0.1	0.9	0.1	0.5	0.0	1.0	0.1	0.6	0.0	0.9	0.1
E	0.8	0.1	***0.4***	***0.1***	1.0	0.0	0.9	0.0	0.7	0.0	1.0	0.1	0.8	0.0	1.0	0.1	0.6	0.1	1.1	0.0	0.5	0.0	0.7	0.1

BC2-NbR106E

	P		E		R		K		A		A		V		S		H		W		Q		Q	
	Ratio	error	Ratio	error	Ratio	error	Ratio	error	Ratio	error	Ratio	error	Ratio	error	Ratio	error	Ratio	error	Ratio	error	Ratio	error	Ratio	error
A	0.2	0.0	0.2	0.1	1.0	0.1	0.4	0.1	***0.2***	***0.1***	***0.1***	***0.0***	0.5	0.0	0.1	0.0	0.3	0.0	1.1	0.1	0.3	0.1	0.1	0.0
G	0.5	0.0	0.4	0.1	1.0	0.1	0.9	0.1	0.4	0.1	0.8	0.1	0.7	0.0	0.8	0.1	0.7	0.0	1.2	0.2	0.4	0.1	0.2	0.0
V	0.3	0.0	0.1	0.0	0.5	0.0	0.1	0.0	0.1	0.0	0.3	0.0	***0.2***	***0.0***	0.2	0.0	0.5	0.0	1.0	0.1	0.2	0.1	0.1	0.0
I/L 1	0.6	0.1	0.1	0.0	0.9	0.2	0.3	0.1	0.1	0.0	0.7	0.1	0.1	0.0	0.5	0.0	0.4	0.0	1.0	0.1	0.2	0.1	0.1	0.0
I/L 2	0.5	0.2	0.2	0.1	0.9	0.2	0.5	0.0	0.1	0.0	0.8	0.1	0.2	0.0	0.9	0.1	0.2	0.0	1.0	0.0	0.4	0.2	0.1	0.0
M	0.4	0.1	0.1	0.0	0.9	0.1	0.3	0.1	0.1	0.0	1.0	0.1	0.3	0.0	1.0	0.1	0.2	0.0	1.1	0.1	0.2	0.1	0.2	0.0
P	***0.1***	***0.0***	0.8	0.1	1.0	0.1	1.0	0.1	0.2	0.0	1.1	0.1	0.9	0.0	1.0	0.1	0.9	0.1	1.1	0.1	0.9	0.1	0.1	0.0
F	0.3	0.0	0.2	0.0	1.0	0.1	0.6	0.1	0.1	0.0	1.0	0.1	0.2	0.0	0.9	0.1	0.2	0.0	1.0	0.1	0.1	0.1	0.3	0.1
W	0.2	0.0	0.1	0.0	1.1	0.2	0.7	0.1	0.0	0.0	1.0	0.1	0.2	0.0	0.9	0.1	0.1	0.0	***0.1***	***0.0***	0.1	0.0	0.2	0.0
S	0.4	0.0	0.2	0.0	0.8	0.1	0.6	0.1	0.2	0.0	0.6	0.0	0.3	0.0	***0.1***	***0.0***	0.4	0.0	1.2	0.2	0.2	0.1	0.1	0.0
T	0.6	0.1	0.1	0.0	0.6	0.0	0.5	0.1	0.1	0.0	0.3	0.0	0.2	0.0	0.1	0.0	0.4	0.0	1.1	0.1	0.2	0.1	0.1	0.0
N	0.7	0.1	0.2	0.1	0.9	0.0	0.8	0.1	0.3	0.1	1.0	0.1	0.4	0.0	0.5	0.1	0.2	0.0	1.2	0.1	0.2	0.1	0.1	0.0
Q	0.6	0.1	0.1	0.0	0.8	0.0	0.2	0.0	0.2	0.0	1.0	0.1	0.4	0.0	0.9	0.1	0.3	0.0	1.2	0.2	***0.3***	***0.1***	***0.1***	***0.0***
Y	0.2	0.0	0.1	0.0	0.9	0.1	0.3	0.1	0.1	0.0	1.0	0.1	0.2	0.0	0.9	0.1	0.1	0.0	1.0	0.0	0.1	0.0	0.2	0.0
C*	0.2	0.0	0.2	0.1	0.9	0.1	0.2	0.0	0.1	0.0	0.1	0.0	0.3	0.0	0.1	0.0	0.3	0.0	1.1	0.1	0.2	0.1	0.1	0.0
K	0.3	0.1	0.1	0.0	0.3	0.0	***0.1***	***0.0***	0.0	0.0	0.9	0.1	0.1	0.0	0.9	0.1	0.2	0.0	1.3	0.1	0.1	0.1	0.0	0.0
R	0.2	0.1	0.1	0.0	***0.1***	***0.0***	0.1	0.0	0.0	0.0	0.7	0.3	0.1	0.0	0.9	0.1	0.1	0.0	1.3	0.2	0.1	0.0	0.0	0.0
H	0.4	0.0	0.1	0.0	0.5	0.0	0.2	0.0	0.2	0.0	1.0	0.1	0.3	0.0	0.9	0.1	***0.2***	***0.0***	1.2	0.2	0.2	0.1	0.1	0.0
D	0.8	0.1	0.2	0.1	1.0	0.0	1.0	0.1	0.9	0.0	1.0	0.1	0.9	0.0	1.0	0.1	0.4	0.0	1.1	0.1	0.5	0.1	0.8	0.1
E	0.8	0.1	***0.2***	***0.1***	1.0	0.0	0.9	0.1	0.8	0.0	1.1	0.1	0.9	0.0	0.9	0.1	0.5	0.0	1.2	0.1	0.4	0.1	0.6	0.0

Supplementary methods

Expression plasmids

For bacterial expression of C-terminal BC2-tagged GFP (GFP_{BC2T}) we used pEGFP-C1 (Clontech) as template. The sequence encoding GFP_{BC2T} was amplified by polymerase chain reaction (PCR) using the oligonucleotide primers GFP_{BC2T}_for (5´-GCA CCA TGG ATG GTG AGC AAG GGC GAG G-3´) and GFP_{BC2T}_rev (5‘-GAC GTC GAC TTA CTG CTG CCA GTG ACT AAC A-3´). The PCR fragment was cloned into the NcoI/SalI restriction sites of pTRC2A (Life Technologies). For expression of the C-terminal BC2-tagged GFP with a N-terminal His_6-tag (His_6-GFP_{BC2T}) the sequence encoding GFP_{BC2T} was amplified by PCR using the oligonucleotide primers His_6-GFP_{BC2T}_for (5´- CAG GGA TCC GAG TGA GCA AGG GC -3´) and His_6-GFP_{BC2T}_rev (5´- CAG GGT ACC TTA CTG CTG CCA GTG ACT AA -3´). The PCR fragment was cloned into BamHI/KpnI restriction sites of pRSET B (Invitrogen) adding an N-terminal His_6-tag.

For mammalian expression of C-terminal BC2-tagged GFP we used pEGFP-C1 as template. The sequence encoding the mammalian $eGFP_{BC2T}$ construct was amplified by PCR using the oligonucleotide primers $eGFP_{BC2T}$_for (5´- AAG CTA GCG CTA CCG GTC GCC ACC ATG -3´) and $eGFP_{BC2T}$_rev (5´- AAG GTA CCT TAT TGC TGC CAG TGA CTA ACA GCC GCT TTT CTG TCT GGC TTG TAC AGC TCG TC -3´). The PCR fragment was cloned into the NheI/KpnI site of the pEGFP-C1 vector.

For mammalian expression of N-terminal BC2-tagged GFP ($_{BC2T}$GFP) the nucleotide sequence encoding the BC2-Tag (5´- GCT AGC ATG CCC GAT CGT AAG GCT GCG GTC TCT CAT TGG CAA CAG AGA TCT -3´) harboring NheI and BglII restriction sites respectively was synthesized (MWG). Subsequently the tag was cloned into NheI/BglII sites of pEGFP-N1 (Clontech). The obtained plasmid was digested with XhoI and NheI, blunted using the Klenow enzyme (Roche) and re-ligated resulting in the desired construct.

For mammalian expression of mCherry-Vimentin$_{BC2T}$ we used a mCherry-Vimentin construct [1] as template. The nucleotide sequence encoding mCherry-Vimentin$_{BC2T}$ was amplified by PCR using the oligonucleotide primers mCherry-Vimentin$_{BC2T}$_for (5´- AAA AGC TTA GGT GGA GGA GGT TCT TCC ACC AGG TCC GTG TC -3´) and mCherry-Vimentin$_{BC2T}$_rev (5´- AAG GTA CCC TAT TGC TGC CAG TGA CTA ACA GCC GCT TTT CTG TCT GGT TCA AGG TCA TCG TG -3´). The PCR fragment was cloned into the HindIII/KpnI sites of the mCherry-Vimentin vector.

For mammalian expression of GFP-PCNA$_{BC2T}$ we used GFP-PCNA [2] as template. The sequence encoding GFP-PCNA$_{BC2T}$ was amplified by PCR using the oligonucleotide primers GFP-PCNA$_{BC2T}$_for (5´- GTA TGG CTT CGT GGG GAT CCC CG -3´) and GFP-PCNA$_{BC2T}$_rev (5´- GGG GTC TAG ACT AAA GGT ACC CTA TTG CTG CCA GTG ACT AAC AGC CGC TTT TCT GTC TGG AGA TCC TTC TTC ATC CTC -3´). The PCR fragment was cloned into the BamHI/XbaI restriction sites of GFP-PCNA vector.

Protein production and purification

Expression and purification of the BC2 nanobody was performed as described previously [3]. Expression and purification of wtGFP or modified versions thereof was performed as described previously [4]. Purity of all proteins was evaluated to be at least 95% based upon sodium dodecyl sulfate-polyacrylamide gel electrophoresis (SDS-PAGE) analysis. Protein concentration was spectroscopically determined.

Mass spectrometry analysis of binding specificities with synthetic positional scanning peptide libraries

To investigate the binding specificity of the BC2T and derived mutants peptide libraries for each amino acid position of the sequence PDRKAAVSHWQQ were synthesized with Acetyl- and Amidgroups located at the N-termini or C-termini respectively (Intavis). Precipitation studies

were carried out incubating 60 pmol peptide of a single position library with 2 µl BC2-Nb immobilized on agarose beads. Incubations were performed in 300 µl PBS/0.01% CHAPS for 1 h on a HulaMixer (Life Technologies). Subsequent to a centrifugation step 10 µl supernatant were analyzed in a LC-MS procedure. Peptides were separated using an UltiMate3000 RSLCnano System (Thermo Scientific), composed of a C18 PepMap100 µ-Precolumn (300 µm x 5 mm; particle size: 5 µm; pore size: 100 Å - Thermo Scientific) and a C18 analytical column (Acclaim Rapid Separation LC (RSLC) Column: 150 mm x 5 mm; particle size: 2.2 µm; pore size: 100 Å - Thermo Scientific). A step gradient was applied starting at 8% and ending after 20 min at 30% eluent B (80% acetonitrile, 20% H_2O, 0.1% formic acid). Peptides were analyzed using a FULL-MS-strategy detected by a Q Exactive Plus mass spectrometer (Thermo Scientific). As maximal injection time 100 ms were chosen while setting the AGC target to 3E6. The resolution was set to 70.000. Half-maximal signal areas were referenced to control precipitation approaches using a GFP-specific nanobody immobilized on agarose beads. All experiments were done in triplicates.

Cell culture and transfection

HEK293T and HeLa cells were cultivated in DMEM (high glucose, pyruvate) supplemented with 10% FCS, 2 mM L-glutamine and Pen Strep (all from Gibco, Life Technologies). Cells were cultivated at 37°C in a humidified chamber with a 5% CO_2 atmosphere and were trypsinized for passaging. To generate DNA/PEI complexes for transient transfection in P100 dishes, 24 µg DNA were mixed with 108 µl polyethyleneimine (PEI, Sigma Aldrich) prediluted with 750 µl Opti-MEM (Gibco, Life Technologies) and incubated for 10 min at RT. For transfection of cells in a 96-well plate, 200 ng of DNA and 1.5 µl of PEI was used.

Generation of soluble protein fraction from bacterial cells

Pellets of *E.coli* cells expressing GFP or GFP_{BC2T} derived from 1 L culture were homogenized for 90 min at 4°C in 500 µl PBS containing 0.1 mg/ml lysozyme, 5 µg/ml DNaseI, 50 µg/ml PMSF and 1x protease inhibitor mix B (Serva) followed by sonication (10 x 10 sec pulses). After a centrifugation step (10 min at 18.000 x g, 4°C), the soluble protein fraction was transferred into a new cup and the protein concentration of each lysate was determined using Coomassie Plus according to the manufacturer's protocol (Thermo Fisher Scientific).

Surface plasmon resonance

The affinity measurements of the BC2 nanobody and the indicated mutants thereof to GFP_{BC2T} were performed using surface plasmon resonance spectroscopy with a Biacore 3000 instrument (GE-Healthcare). GFP_{BC2T} was covalently coupled on dextran fibers of a CM5 sensorchip (GE Healthcare) to a response level of 500 RU. One flow cells was activated and blocked in the absence of protein to determine background, another was loaded with untagged GFP as a control against unspecific binding. For kinetic measurement, six concentrations ranging from 7.8125 nM to 250 nM of either BC2-Nb, BC2-Nb_{R106S} or BC2-Nb_{R106E} were injected. Each measurement was done in duplicates. As running/dilution buffer 10 mM HEPES pH 7.4, 150 mM NaCl, 0.5% surfactant Tween was used. Measurements were performed at 25 °C. For the association of BC2-Nb a flow rate of 50 µl/min for 15 s and for the dissociation a flow rate of 50 µl/min for 600 s was applied. The regeneration was induced by injection of 23 µl regeneration solution at a flow rate of 30 µl/min. As regeneration solution 10 mM glycine-HCl pH 2.0 was used. The data was evaluated using the software Bia evaluation 4.1 and the 1:1 Langmuir binding model with mass transfer.

SDS PAGE and immunoblotting

Denaturing polyacrylamide gel electrophoresis (SDS-PAGE) was performed according to standard procedures. Protein samples were boiled in 2x SDS-sample buffer (60 mM Tris/HCl,

pH 6.8; 2% (w/v) SDS; 5% (v/v) 2-mercaptoethanol, 10% (v/v) glycerol, 0.02% bromphenole blue). For immunoblotting proteins were transferred on nitrocellulose membranes (Bio-Rad Laboratories).

Antibodies

For immunoblotting the following primary antibodies were used: anti-GFP clone 3H9 (ChromoTek), anti-PCNA clone 16D10 (ChromoTek), anti-Vimentin clone V9 (Sigma Aldrich), anti-GAPDH (abcam), anti-β-catenin clone 14 (BD-Biosciences). For detection fluorophore-labeled species-specific secondary antibodies (Alexa-647, goat-anti-rabbit, goat-anti-rat; goat-anti-mouse Life Technologies) were used. Blots were scanned on the Typhoon-Trio laser scanner (GE Healthcare).

Immunoprecipitation at harsh conditions

For each condition, 50 µl bead-slurry were mixed with 100 µl of soluble protein fraction from *E.coli* lysate (c=1 mg/ml) and 150 µl of a solution containing either a detergent or a chaotropic agent, resulting in a final concentration as indicated below. As chaotropic agents we used urea (0 M, 1 M, 2 M, 4 M) and guanidinium chloride (0 M, 0.375 M, 0.75 M, 1.5 M, 3 M) and as a detergent we used SDS (0%, 0.1%, 0.5%, 1%, 2%). After incubation on an end-over-end rotor for one hour at 4 °C and a centrifugation step (2 min, 2500 x g, 4 °C) the supernatants were discarded and the remaining beads were washed twice in PBS before boiling in 50 µl 2 x sample buffer. 10% of each bead bound (B) fraction were analyzed by SDS-PAGE and immunoblotting using an anti-GFP antibody.

Elution of bound BC2-tagged protein

For the elution experiments 40 µl of BC2 nanotrap (slurry) were incubated with 400 µl soluble protein extract (c = 1mg/ml) derived from *E.coli* cells expressing GFP_{BC2T} for 1 h at 4°C on an

end-over-end rotor. After a centrifugation step (2500 x g, 4 °C, 2 min) the supernatant was discarded and the beads were washed four times in ice-cold PBS including a cup change after the second washing step. Subsequently, beads were pelleted and incubated with 80 µl of the indicated elution conditions for 15 min at RT. The following elution conditions have been tested: peptide elution: 0 mM, 0.01 mM, 0.1 mM or 1 mM BC2-peptide dissolved in in 0.2 M Tris/Cl pH 7.4, 150 mM NaCl; acidic elution: 0.2 M Glycine-HCl adjusted to pH 1, pH 2 or pH 3; alkaline elution: 0 mM, 1 mM, 10 mM or 100 mM NaOH; elution with sodium thiocyanate: 0 M, 1 M, 2 M, or 3 M NaSCN. The eluates were collected and boiled in 1 x SDS containing sample buffer and the remaining beads were washed twice in PBS before boiling in 40 µl 2 x sample buffer. 10% of each elution (release, R) and bead bound (bound, B) fraction was analyzed by SDS-PAGE and immunoblotting using an anti-GFP antibody.

Fluorescence spectroscopy.

Fluorescence assays were performed by scanning a 96 well microplate (Nunc) on a Typhoon Trio (GE Healthcare Life Sciences, excitation: 488 nm, emission filter settings: 520 nm BP 40).

References

1 Maier, J., Traenkle, B. & Rothbauer, U. Real-time analysis of epithelial-mesenchymal transition using fluorescent single-domain antibodies. *Sci Rep* **5**, 13402 (2015).

2 Leonhardt, H. et al. Dynamics of DNA replication factories in living cells. *J Cell Biol* **149**, 271-80 (2000).

3 Traenkle, B. et al. Monitoring interactions and dynamics of endogenous beta-catenin with intracellular nanobodies in living cells. *Mol Cell Proteomics* (2015).

4 Kirchhofer, A. et al. Modulation of protein properties in living cells using nanobodies. *Nat Struct Mol Biol* **17**, 133-8 (2010).
